# Cryopreserved platelets in bleeding management in remote hospitals: A clinical feasibility study in Sweden

**DOI:** 10.3389/fpubh.2022.1073318

**Published:** 2023-01-20

**Authors:** Agneta Wikman, Beatrice Diedrich, Karl Björling, Per-Olof Forsberg, Anna-Maria Harstad, Ragnar Henningsson, Petter Höglund, Hans Sköld, Lars Östman, Per Sandgren

**Affiliations:** ^1^Clinical Immunology and Transfusion Medicine, Karolinska University Hospital, Stockholm, Sweden; ^2^Department of Clinical Science, Intervention and Technology (CLINTEC), Karolinska Institutet, Stockholm, Sweden; ^3^Department of Anaesthesiology and Intensive Care, Visby Hospital, Visby, Sweden; ^4^Department Laboratory Medicine, Central Hospital of Karlstad, Karlstad, Sweden; ^5^Department of Anaesthesiology and Intensive Care, Central Hospital of Karlstad, Karlstad, Sweden; ^6^Center for Hematology and Regenerative Medicine, Department of Medicine, Huddinge Karolinska Institutet, Stockholm, Sweden; ^7^Department of Anaesthesiology and Intensive Care, Torsby Hospital, Torsby, Sweden

**Keywords:** BLEE-bleeding, platelets (PLT), blood transfusion, urban or rural, cryopreservation, preparedness activities

## Abstract

**Background:**

Balanced transfusions, including platelets, are critical for bleeding patients to maintain hemostasis. Many rural hospitals have no or limited platelet inventory, with several hours of transport time from larger hospitals. This study aimed to evaluate the feasibility of using cryopreserved platelets that can be stored for years, in remote hospitals with no or limited platelet inventory.

**Material and methods:**

Three remote hospitals participated in a prospective study including adult bleeding patients where platelet transfusions were indicated. Cryopreserved platelets were prepared in a university hospital, concentrated in 10 ml, transported on dry ice, and stored at −80°C at the receiving hospital. At request, the concentrated platelet units were thawed and diluted in fresh frozen plasma. The indications, blood transfusion needs, and laboratory parameters pre- and post-transfusion, as well as logistics, such as time from request to transfusion and work efforts in preparing cryopreserved platelets, were evaluated.

**Results:**

Twenty-three bleeding patients were included. Nine patients (39%) were treated for gastrointestinal bleeding, five (22%) for perioperative bleeding, and four (17%) for trauma bleeding. The transfusion needs were 4.9 ± 3.3 red blood cell units, 3.2 ± 2.3 plasma units, and 1.9 ± 2.2 platelet units, whereof cryopreserved were 1.5 ± 1.1 (mean ± SD). One patient had a mild allergic reaction. We could not show the difference in laboratory results between pre- and post-transfusion of the cryopreserved units in the bleeding patients. The mean time from the order of cryopreserved platelets to transfusion was 64 min, with a range from 25 to 180 min.

**Conclusion:**

Cryopreserved platelets in remote hospitals are logistically feasible in the treatment of bleeding. The ability to have platelets in stock reduces the time to platelet transfusion in bleeding patients where the alternative often is many hours delay. Clinical effectiveness and safety previously shown in other studies are supported in this small feasibility study.

## Introduction

The availability of blood products in small remote hospitals is often limited and without reserve capacity. The blood inventory is planned according to normal usage, aiming for low wastage. With long transport time to larger hospitals, these hospitals occasionally treat patients with large bleeding, due to trauma, obstetric bleeding, gastrointestinal bleeding, and unexpected bleeding in surgical procedures. If massive bleeding occurs, the blood stock can quickly be emptied. The most critical blood product in rural settings is platelets. Platelets have a short shelf time of 5–7 days and are a product with high cost. In massive bleeding cases a small stock of platelets, often 1-2 units is insufficient, but results in high wastage most of the time (1). As a result, many smaller hospitals have no platelets stored locally, leading to several hours of delay before treatment. In treatment guidelines for bleeding, the early use of blood products is recommended and has improved survival (2, 3). Platelets are crucial for hemostasis in bleeding patients (4) resulting in reduced use of red blood cells and plasma products, which is important in hospitals with limited blood inventory (5). In addition, many patients are on life-long treatment with anti-coagulant and platelet inhibitory drugs, with an increased risk of bleeding, as a consequence. Cold-stored platelets with up to 2 weeks of storage time have been evaluated in the treatment of bleeding (6). This may facilitate the logistics but probably not enough to ensure a supply in occasional bleeding.

Cryopreserved platelets extend platelet shelf life and the upper limit for storage has currently been set to 1 year in the European Guide to the preparation, use, and quality assurance of blood components (EDQM) (7). However, studies indicate an extended shelf life of up to 12 years (8), which will make it possible to have a platelet stock in remote hospitals. Cryopreserved platelets have been used on special indications since the 1970s, and the early use was most often in refractory hematologic patients (9). The freezing protocols have been evaluated and improved, and the standard is now 5–6% dimethyl sulfoxide (DMSO) and dilution in plasma. The platelet recovery after thawing varies in different reports but is most often 50–70% (10, 11). Cryopreserved platelets have been extensively evaluated *in vitro* after thawing and are judged functional and hemostatically effective (12). In contrast, the recovery rate is 60–70% on average, and a comprehensive *in vitro* characterization of the cryopreserved platelets before clinical studies has been presented in recent publications. We have analyzed and evaluated the ultrastructural and functional effects (13), hemostatic responsiveness and the release of biological response modifiers (14), cryopreservation of platelets using different freezing rate protocols (14), and the potential effects of cryopreservation on the interaction with different plasma derivates (15). Cryopreserved platelets are preactivated and adhere to activated endothelium and are not recommended as prophylactic transfusions.

Frozen platelets have been used in military trauma casualties, with good clinical effects and no reported adverse reactions (16, 17). The first clinical randomized trial was published in 1999, 73 cardiac surgery patients received cryopreserved or standard fresh platelets (18). The patients treated with cryopreserved platelets needed significantly fewer blood transfusions and no adverse effects were seen. Cryopreserved vs. Liquid Platelets (CLIPs), a pilot study in perioperative bleeding in Australia, and a pilot study from New Zealand were recently published (19, 20). Both studies support the safety of cryopreserved platelets. Cryopreserved platelets are approved and described in the EDQM (7).

In Scandinavia, as well as in Australia and New Zealand, and reported from the USA and Canada (21), there are many rural hospitals with limited blood stock and no platelets available.

The primary aim of this prospective observational study was to evaluate the challenges with logistics, in remote hospitals with no or limited platelet inventory, with the secondary aim of assessing clinical demand, indications, and the effect of using cryopreserved platelets in bleeding. This feasibility study lays the foundation for further investigations to more comprehensively assess the hemostatic effectiveness of cryopreserved platelets in remote locations.

## Materials and methods

### Study design

From August 2019 to December 2021, three hospitals participated in a prospective study including adult bleeding patients where platelet transfusions were indicated. When a platelet unit was ordered, a frozen platelet could be chosen. Two of the hospitals normally also had fresh platelets in stock, but one of the hospitals had no other platelets. If there were fresh platelets available, we left it up to the clinician to decide. We did not know the challenges and time it would be to prepare platelets in a small laboratory, and did not want delay transfusion in a bleeding patient.

### Study hospitals

Two hospitals with remote locations participated. One larger regional hospital participated in the last period, out of interest to evaluate frozen platelets as a backup stock.Visby Hospital is a general hospital located on an island with limited transport opportunities in emergency situations. The hospital has ~140 beds, offering healthcare to more than 60,000 inhabitants, which would double during the summertime. The hospital has elective and trauma surgery, an emergency department, an intensive care unit (ICU), and a delivery ward with 500–600 deliveries annually. The normal blood inventory is ~140 red blood cell (RBC) units, 100 fresh frozen plasma (FFP) units, and 2–4 units of platelets. In 2019, more than 2000 RBC units, close to 200 plasma units, and 200 platelet units were transfused.

Torsby Hospital is located in a small city close to the border with Norway, with a drive time of 1.5–2 h to a larger hospital. The hospital has 90 beds in three wards, an ICU with four beds, and an intensive medical department with three beds. The hospital serves a population of 10,000 with an emergency department, general and orthopedic surgery, internal medicine, and gynecology, but not deliveries. The inventory is 66 RBC units, 18 plasma units (3 liquid), and no platelets. In 2019, the use of blood included ~800 RBC units, 50 plasma units, and 12 units of platelets.

Karlstad Hospital is a 450-bed regional hospital offering specialized care in an area with long distances in the western part of the country. Annually 20,000 surgical procedures and 2,700 deliveries are done. Helicopter and land-based ambulances have many acute means of transport.

The helicopter carries two units of whole blood. The normal blood inventory is 270 RBC units, 100 plasma units (6 liquid), and 10 platelet units. In 2019, 8500 RBC units, 650 plasma units, 1200 platelet units, and 18 whole blood units were transfused.

### Inclusion of patients and evaluation of frozen platelets

During 2019–2021, consecutive bleeding patients in the two small rural hospitals were included. In 2021, a third regional hospital was added and included three additional patients. Frozen platelets were used in clinical indications in bleeding adult patients. They were not used for prophylactic transfusions. Inclusion of patients, clinical effect, as subjectively assessed by the treating anesthesiologist or surgeon as improved clotting or stopped bleeding, and laboratory parameters pre- and post-transfusion, were all documented in a protocol, Appendix 1. The involved staff evaluated the logistics, including time and work efforts, using a laboratory protocol, Appendix 2. The transfused blood components and laboratory data were collected from the laboratory systems.

### Preparation, transportation, and thawing of cryopreserved platelets

For cryopreservation, a total of 30 newly produced platelet units derived from pooled buffy coats were transferred to a freezing bag (Macopharma) using a sterile connection device (Terumo BCT, Lakewood, CO, USA). A mixture of 25% DMSO/NaCl (50 ml) was then sterile docked, and the solution was added to the platelet concentrates. After centrifugation at 1,200 *g* for 10 min, as much supernatant as possible was removed, leaving 0.5 ml freezing medium in ~10 mL of platelets. The freezing bags, containing ~10 ml of platelet (5% DMSO), were immediately frozen in sheet metal boxes using a fast-uncontrolled freezing rate protocol at −80°C. The frozen units were transported on dry ice to the receiving hospitals using a transport courier and immediately placed in a low-temperature freezer at −80°C. When a platelet unit was requested, the cryopreserved platelets were thawed in a thawing bath maintained at 37°C for 1 min. The thawed and cryopreserved platelets were then sterile docked and gently reconstituted with compatible fresh thawed plasma to a total volume of 200 ml. The platelet unit was resting, non-agitated, until transfusion.

### Laboratory parameters pre- and post-transfusion

The hospital laboratories performed routine cell count and coagulation tests, including fibrinogen (g/L) (Clauss coagulation photometry), prothrombin time (PT) international normalized ratio (INR), and activated partial thromboplastin time (APTT). Analysis by thromboelastography (TEG) (Haemonetics Corp Boston, Mass. USA) was performed during the study, but not used in routine in the two small hospitals. ROTEM (GmbH, Leipzig, Germany) was used in the largest hospital.

### Statistical analyses

The mean values and standard deviation are given unless otherwise indicated. A *t*-test was assessed to determine the statistical significance of the differences in laboratory values pre- and post-transfusion. The analyses were carried out using GraphPad Prism version 9.0 (121) (GraphPad San Diego CA USA).

### Ethics

The study was approved by the Swedish Regional Ethics Review Boards Dno 2019-00350 and 2020-05579. Signed approval was obtained from the participating centers. In accordance with the ethical approval, informed consent was not required from the patients.

## Results

### Included patients

The study included 23 bleeding patients over the course of more than 2 years, as shown in Table 1. Nine patients (39%) were treated for gastrointestinal bleeding, five (22%) for perioperative bleeding, and four (17%) for trauma bleeding. Twelve (52%) of the patients were included at the smallest hospital, where they had no other platelets in stock, eight at the middle-sized hospital, and three at the larger hospital joining the last period of the study. Cryopreserved platelets were used on all bleeding patients in the smallest hospital, during the study period. In the middle-sized hospital, 518 platelet units were transfused during the study period of which ~130 units were to bleeding patients and the rest to prophylactic transfusions to hematology and oncology patients. Wastage of platelets, due to expiry, was close to 40% in this hospital located on an island. The largest hospital participating in the last 6 months of the study of interest to test the feasibility of cryopreserved platelets as a backup stock included three of 22 bleeding patients.

**Table 1:** Patient characteristics.

**Included patients**	**23**
Male/female	16/7
Age mean ± SD, min, max	64.6 ± 14,3; 27,90
**Diagnosis**	
Gastrointestinal bleeding	9
Trauma bleeding	4
Peroperative bleeding (hysterectomi, pancreactomi, cholecystectomy+liver, liver, mesenterial)	5
Bleeding due to septicemia, DIC	2
Bleeding due to thrombocytopenia	2
Bleeding due to sinus thrombosis and anti-coagulant therapy	1

### Laboratory data, blood transfusions, and complications

**Figure 1:**
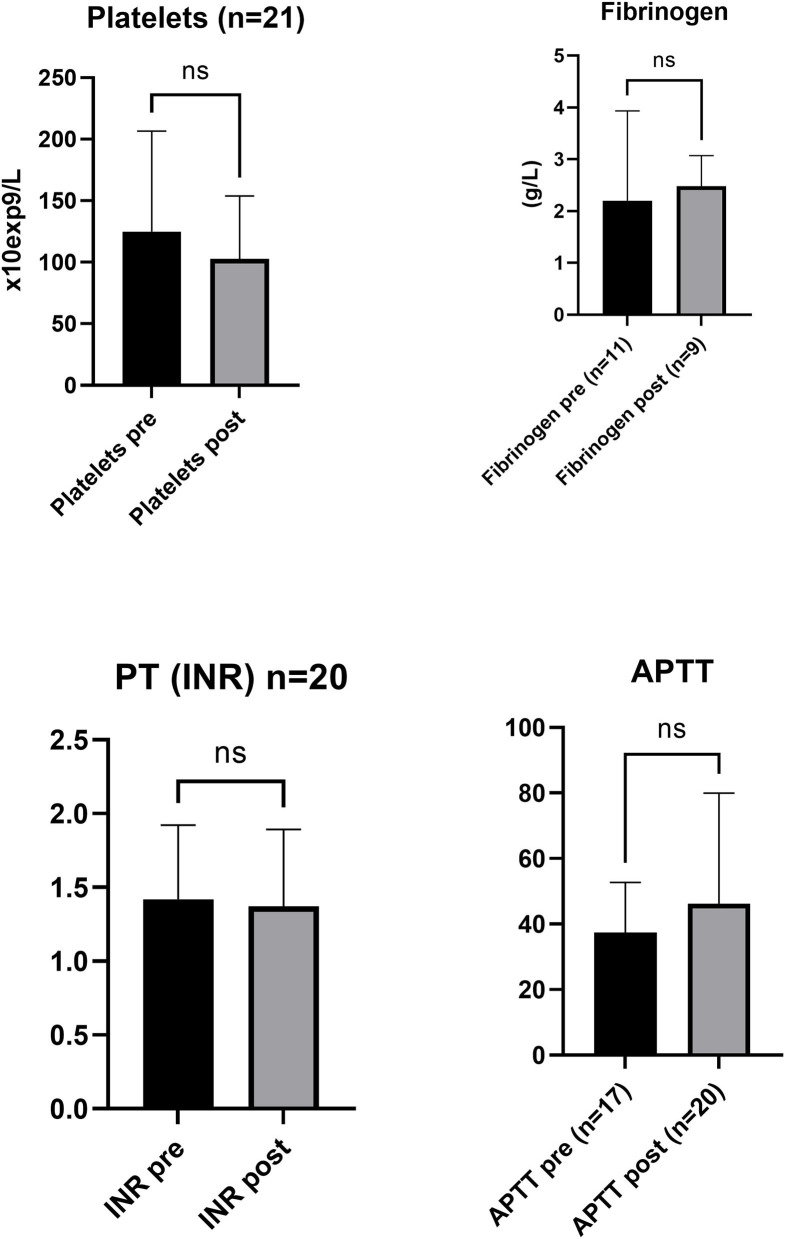
Cell count, concentration of coagulation factor, and viscoelastography properties pre- (Base) and post-treatment. Cell count, fibrinogen (g/L) (Clauss coagulation photometry), prothrombin time (PT) international normalized ratio (INR), and activated partial thromboplastin time (APTT) analysis presented pre-treatment and post-treatment of platelet transfusion. Bar graphs show mean ± SD for (*n* = ) pre-treatment and post-treatment of platelet transfusion. ns indicates non significant difference compared to pre-treatment.

**Figure 2:**
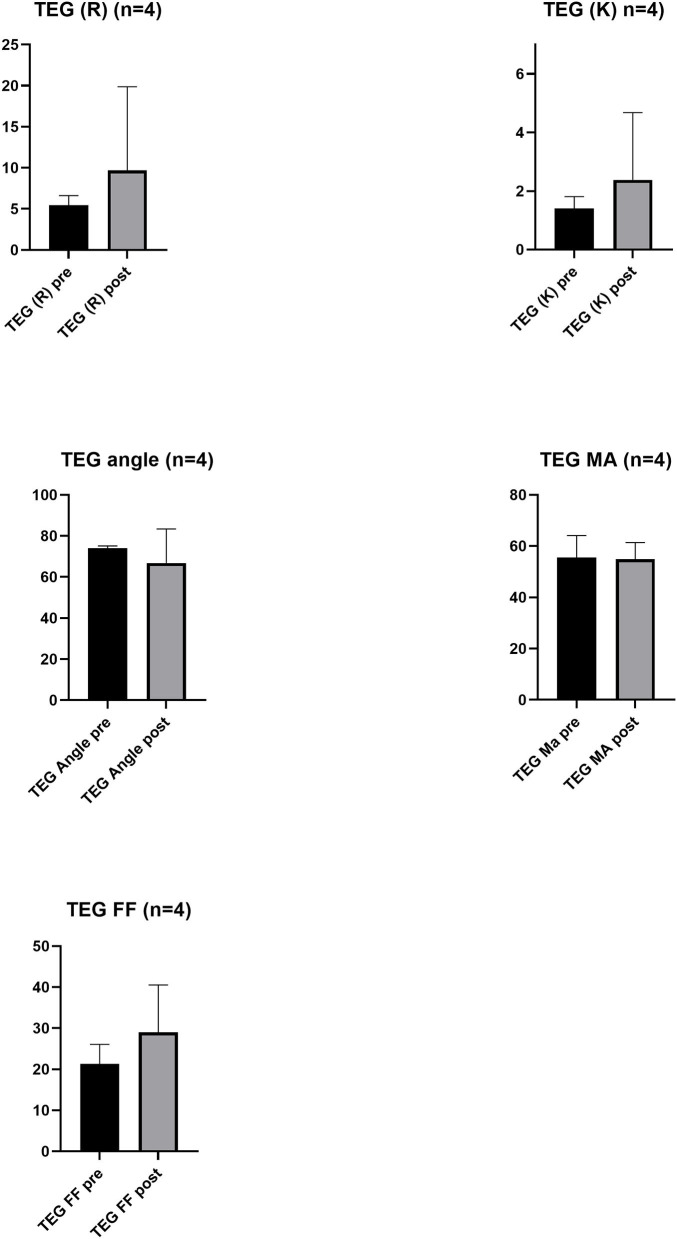
Viscoelastography properties pre- (Base) and post-treatment. Thromboelastography (TEG) output for reaction rate (R) (min), kinetics time (K) (min), angle (α) (degrees), maximum amplitude (MA) (mm), and functional fibrinogen (FF). Bar graphs show mean ± SD for *n* = 4 pre-treatment and post-treatment of platelet transfusion.

In 6 of the 23 patients, the platelets were assessed to have a visible effect (improved clotting or stopped bleeding), in nine, there was no visible effect, and in eight, there was no comment in the protocol by the treating clinician (Appendix 1). There was no significant statistical difference in laboratory values, referring to platelet count, fibrinogen, INR, APTT, and TEG (used in one hospital) and ROTEM (used in one hospital, data not shown) parameters, pre- and post-transfusion (Figures 1, 2). The collection of samples was not consistently done according to the protocol, resulting in missing data. The time of the blood sampling in relation to the transfusion of the platelet unit, other blood components, and fibrinogen was unclear, making the laboratory results difficult to interpret. Viscoelastic testing was not part of the normal routine in the two smaller hospitals, and was then not at all used in the smallest hospital and not consistently used in emergent situations in the other hospital. In the largest hospital, ROTEM was used. The results from viscoelastic testing were few and difficult to interpret for the same reason as other laboratory analyses. In total, 14 of the 23 patients were transfused with only one frozen platelet unit, six patients were transfused with two frozen platelet units, two patients were also treated with one unit of fresh platelets, and one trauma patient was treated with five fresh and six frozen platelet units, in mixed order. Two patients did not survive, one was transported to a university hospital due to surgical complications and died there, and one died in septic shock. The transfusion needs were 4.9 ± 3.3 red blood cell units, 3.2 ± 2.3 plasma units, and 1.9 ± 2.2 platelet units, whereof cryopreserved were 1.5 ± 1.1 (mean ± SD) (Table 2). There was a mild allergic reaction in one patient, possibly due to the plasma unit. No other complications associated with the platelet transfusions were seen. The length of stay in the hospital was 11.8 ± 14.4 (mean ± SD), with a range of 1–46 days.

**Table 2:** Outcome.

**Blood transfusions**	
Red blood cells units mean ± SD; min,max Plasma units mean ± SD; min,max	4.9 ± 3.3; 0, 13 3.2 ± 2.3; 0, 9
Platelet units total mean ± SD; min, max Platelet units cryopreserved mean ± SD;min, max	1.9 ± 2.2; 1, 11 1.5 ± 1.2; 1, 6
**Complications**	
Reoperation	1
Abscess Transfusion reactions Mortality **Length of stay** mean ± SD; min, max	1 1 2 11.8 ± 14.4; 1, 47

### Storage and logistics

The mean time from the order of platelets to transfusion was 64 min, with a range from 25 to 180 min (Table 3). In the two rural hospitals in our study, there was only one biomedical scientist working night and weekends, responsible for all laboratory analyses and blood product requests, that is, blood counts, coagulation tests, blood type, and type and screen, as well as requested blood units, in bleeding patients. After initial training and practice, the opinion was that the procedure to prepare the cryopreserved platelets was simple. Tested with trained personnel, the procedure was done in <15 min, provided thawed plasma is available. If platelets are not locally available, the transport of fresh platelets to the smallest hospital in the study is ~2 h from the time of order. To the hospital on the island, transport is often not possible for many hours and is available only in the daytime. Cryopreserved platelets stored locally can be transfused within 30–60 min from the order.

**Table 3:** Clinical effect and logistics.

**Clinical opinion**	
Visible effect	6
No visible effect No comment	9 8
**Logistics time from order to delivery (min)**	
mean ± SD; min, max	66.0 ± 37.2; 25, 180

## Discussion

In this study, in three remote hospitals, we show the feasibility of using cryopreserved platelets in the treatment of bleeding. The primary aim of the study was to evaluate the logistics of using cryopreserved platelets in acute bleeding, and the secondary aims were to assess indications and clinical effects. The preparation of the cryopreserved platelets and distribution on dry ice from a university hospital to the receiving hospital and the local storage in a −80°C freezer were performed without difficulties. For the local preparation of the platelet units, the staff needs training and instructions, and the time from request to transfusion varied between 25 and 180 min in the study, but with trained personnel, it is done in 15–30 min. The indications for platelet transfusions can vary depending on the level of healthcare offered in hospitals, and in our study reflecting general care, the indications were due to gastrointestinal bleeding, complications in elective surgery, and trauma, which are probably representative of smaller hospitals. Two of the hospitals have also obstetric wards, but no obstetric patients were included during the study period. We could not show the difference in laboratory values pre- and post-transfusion, after transfusion of one to two platelet units in bleeding patients, which was expected. Samples were not collected consistently pre- and post-transfusion, and the administration of red blood cells, plasma, and fibrinogen in relation to blood sampling was not documented, making the results difficult to interpret. The clinical assessment was that the platelets were hemostatic, supported by an adequate ratio of platelets to red cells and plasma before bleeding was controlled. No one died of bleeding. There was one mild allergic reaction, and no other adverse reactions were seen, supporting previously published data on safety. The inclusion of patients was slower than expected and the planned protocol was often violated, resulting in missing data and reflecting the challenges with research studies in small hospitals with limited staff resources and rotating physicians. Despite that, the subjective opinion from the clinicians was that the cryopreserved platelets were hemostatic and offered an increased therapeutical arsenal and redundancy at the hospital.

Platelets are important in the treatment of bleeding but are often not available or of insufficient supply in many rural hospitals in our country as well as other countries and thus bleeding patients may not be optimally treated (2, 21). Initially, we included patients in two remote hospitals. One of the hospitals has normally no platelets in stock, due to high wastage and high costs. The annual usage is estimated to be 10–20 platelet units, the majority was prophylactic transfusions. Transport time of platelets is 2 h from the nearest larger hospital. The other hospital is located on an island in the Baltic Sea, with challenging transportation. The hospital has normally 2–4 platelet units in stock, resulting in ~40% wastage but often deficiency in acute bleeding situations. A third regional hospital expressed interest to participate and evaluate cryopreserved platelets as a backup to a normal stock of 10 units, which may be important in mass causality situations.

The quality and *in vitro* function of cryopreserved platelets have been extensively studied by us and others, in many publications (10, 11, 15). The platelets are activated and affected in the freezing and thawing procedures, but still contain adequate hemostasis function when measured by viscoelastography (13, 15). Cryopreserved platelets have been used clinically on special indications since the 1970s (9). In an early clinical study by Khuri et al. from 1999, where bleeding patients were randomized to either cryopreserved or standard platelets, the conclusion was that cryopreserved platelets were superior to liquid platelets in reducing blood loss and the need for blood transfusions (18). Despite the promising results, cryopreserved platelets have not been widely implemented, but recently, there is a growing interest both for military use and in remote hospitals where transportation and logistics are complicated. Until now many hundreds of cryopreserved platelet units have been transfused with safe results (20). There is no increased incidence of adverse reactions shown. The final results from ongoing prospective randomized clinical trials in Australia and New Zealand addressing non-inferiority to standard platelets will be important (19, 20).

Limitations of this study include the study being done in small hospitals with limited staff resources and a limited number of bleeding patients. The initial aim included laboratory analyses pre- and post-transfusion, which often were missed. Analysis by TEG was offered but not used in one of the hospitals and rarely in the other. We were careful not to delay platelet transfusions to bleeding patients, so there were no recommendations to prioritize cryopreserved platelets more than in the hospital where no other platelets were available. Patients were included based on the availability of fresh platelets, which were often prioritized, by the working clinicians, In addition, the information of the study to deputy clinicians, common in rural hospitals, was often insufficient. Thus, the clinical effects of cryopreserved platelets cannot be evaluated from our study, but nothing is contradicting previous results reporting effect and safety (9, 17, 18, 20), from larger studies.

We conclude that cryopreserved platelets in remote hospitals are feasible and that cryopreserved-thawed platelets may theoretically be transfused within 30 min and were most often transfused in an hour in our small study. The ability to have platelets in stock increases the safety for bleeding patients, where the alternative may be delayed treatment with many hours of transport of platelets from a larger hospital. In addition, cryopreserved platelets can be used as a backup stock in larger hospitals, e.g., during long weekends when it is difficult to predict the need, in mass causality situations, and in military preparedness. There is potential to further improve the freezing and thawing procedures and to increase the storage time from today's 1 to 4 years to a longer time, that is, 12 years reported in a recent study (8). Our study describes the logistics of using cryopreserved platelets in small remote hospitals and supports the potential of having platelets available in all hospitals occasionally treating bleeding patients. However, preparation and thawing procedures can be further improved, and additional studies of *in vitro* and *in vivo* performance of cryopreserved platelets are warranted.

## Data availability statement

The original contributions presented in the study are included in the article/Supplementary material, further inquiries can be directed to the corresponding author.

## Ethics statement

The studies involving human participants were reviewed and approved by Swedish Regional Ethics Review Boards nos. 2019-00350 and 2020-05579. Written informed consent for participation was not required for this study in accordance with the national legislation and the institutional requirements.

## Author contributions

AW, LÖ, KB, HS, and P-OF designed the study. LÖ, KB, HS, A-MH, and RH included the patients. BD, PH, and P-OF supported the logistics in the lab. PS produced the cryopreserved platelets. AW and PS wrote the first draft of the manuscript. All authors contributed to the writing and approved the final version of the manuscript.
