# Supplementary material for: Cryopreserved platelets in bleeding management in remote hospitals: A clinical feasibility study in Sweden
Source: Front Public Health. 2023 Jan 20;10:1073318. doi: 10.3389/fpubh.2022.1073318 (PMC9894868; doi:10.3389/fpubh.2022.1073318)
Supplement: Supplementary file 1 [file Table_1.DOCX]

**Clinical Study protocol ”Frozen Platelets”**

**Inclusion and evaluation by treating anesthesiologist**

| Patient ID |  | | |
| --- | --- | --- | --- |
| Diagnosis and indication |  | | |
| Date and time of platelet request |  | | |
| Date and time of arrival of platelets |  | | |
| Date and time of transfusion | **Unit 1** | | **Unit 2** |
| Hb, Platelet count before transfusion 4.5 mL EDTA sample |  | |  |
| Hb, platelet count after transfusion 4.5 mL EDTA sample |  | |  |
| TEG/ROTEM before transfusion 4.5 mL citrate sample |  | |  |
| TEG/ROTEM after transfusion  4.5 mL citrate sample |  | |  |
| INR, Fibrinogen, APTT before tranfusion  4.5 mL citrate sample |  | |  |
| INR, Fibrinogen, APTT after transfusion  4.5 mL citrate sample |  | |  |
| Estimated bleeding incl drainage at 24 hours |  | | |
| Number of transfusions RBC/plasma/platelets within 24 hours | RBC | Plasma | Platelets |
| Assessed effect (coagulation, stopped bleeding) Yes/no/comments |  | | |
| Transfusion complication |  | | |
| Postoperative complications |  | | |
| Other comments |  | | |
| Length of stay (LOS) |  | | |
