# Supplementary material for: Cryopreserved platelets in bleeding management in remote hospitals: A clinical feasibility study in Sweden
Source: Front Public Health. 2023 Jan 20;10:1073318. doi: 10.3389/fpubh.2022.1073318 (PMC9894868; doi:10.3389/fpubh.2022.1073318)
Supplement: Supplementary file 2 [file Table_2.DOCX]

**Laboratory Protocol “**Frozen platelets”

| **Patient ID** |  | | |
| --- | --- | --- | --- |
| **Platelet unit 1** | Date and time of request | Date and time of issue | Sign |
| **Platelet unit 2** |  |  | Sign |
| **User opinion**  **Score 1-5 (5 is best)** |  |  | Sign |
|  |  |  | Sign |
| **Comments** | | | |
